# Coffee and Cardiovascular Health: A Review of Literature

**DOI:** 10.3390/nu16244257

**Published:** 2024-12-10

**Authors:** Altaf Farraj, TiJesu Akeredolu, Anisha Wijeyesekera, Charlotte E. Mills

**Affiliations:** Department of Food and Nutritional Sciences, School of Chemistry, Food & Pharmacy, University of Reading, Reading RG6 6AP, UK; a.k.farraj@pgr.reading.ac.uk (A.F.); t.akeredolu@pgr.reading.ac.uk (T.A.); a.wijeyesekera@reading.ac.uk (A.W.)

**Keywords:** coffee, cardiovascular disease, CVD, caffeine, chlorogenic acid, trigonelline, diterpene

## Abstract

Cardiovascular disease is the leading cause of death worldwide and is on the rise. Diet is considered to be a key modifiable risk factor for reducing the incidence of cardiovascular disease. Dietary approaches have proved advantageous for preventing disease morbidity and mortality but tend to focus on fruit, vegetables, fiber, lean protein and healthy fats. Coffee is one of the most popular beverages worldwide but is often surrounded by controversy with regard to its impact on health. This review aims to explore the relationship between coffee consumption and cardiovascular disease. The evidence from observational trials as well as randomized controlled trials is discussed. By focusing on specific bioactive compounds in coffee, potential mechanisms are explored, and future directions of research in the field are considered.

## 1. Introduction

Cardiovascular disease (CVD) is the primary cause of mortality worldwide, responsible for an estimated 17.3 million deaths annually. Projections indicate that this number will rise to over 23.6 million by 2030 [1]. Cardiovascular disease can broadly be defined as a disease that affects the heart or blood vessels; examples of CVD include coronary heart disease, stroke, peripheral arterial disease and aortic disease [2], with additional vascular factors that can increase the risk of CVD (e.g., hypertension or dyslipidemia). The relationship between diet and cardiovascular health is well-established, with dietary habits playing a crucial role in both the prevention and management of CVD, indeed, diet has been identified as a key modifiable risk factor in the reduction in CVD. Dietary patterns such as the Mediterranean diet and the Dietary Approaches to Stop Hypertension (DASH) diet have been extensively studied for their cardiovascular benefits [3]. These diets emphasize fruit and vegetables, wholegrains, lean proteins and healthy fats and have been shown to lower the risk of CVD through various mechanisms, including improved lipid profiles, blood pressure regulation, and anti-inflammatory effects [4]. Understanding the complex interplay between diet and CVD is essential for developing effective nutritional strategies aimed at preventing and managing CVD.

Coffee is a beverage widely consumed globally which has historically been associated with negative health connotations. Indeed, caffeine (caffeinated coffee) is associated with issues in pregnancy, and hence it is recommended to limit intake of caffeine when pregnant [5]. It is now accepted that the historical negative associations observed between coffee and heart health are likely due to confounding factors such as cigarette smoking and sedentary behaviors [6]. More recently coffee is gaining increasing interest for health benefits, including reducing all-cause mortality, cancer risk, neurological, metabolic and liver conditions as well as the role it may play in CVD prevention [7]. The media and social media are interested in the impact of coffee on health, but the messages can be confusing and often conflicting. Confusion may stem from the complexity of coffee research, especially in relating the consumption with health. Here, we discuss the evidence for coffee consumption in relation to CVD risk reduction and disease prevention. In this respect, we have separated the evidence assessing the impact of coffee as a beverage from that looking at specific bioactives (e.g., caffeinated versus decaffeinated coffee). This has seldom been performed and causes a conflation of two different hypotheses. We have, however, tried to explain some of the health effects observed with coffee consumption by considering the potential bioactives within coffee that could play a role.

## 2. Impact of Coffee on Cardiovascular Disease

Since the turn of the century, peer-reviewed publications on coffee have increased rapidly, including those focusing on the impact of coffee on CVD. Historically, the impact of coffee on cardiovascular health was assumed to be negative, probably due to confounding factors, as well as the apparent negative effects of caffeine. However, more recent data seem to contradict those findings, and there is a suggestion that coffee could play a role in disease prevention.

### 2.1. Evidence from Observational Trials

There are a large number of observational trials associating coffee consumption with a reduction in CVD incidence, mortality from CVD, and CVD risk factors. In 2017, two umbrella reviews of observational trials were published which demonstrated that moderate coffee consumption was associated with a reduction in cardiovascular mortality and CVD [7,8]. Since the searches were conducted in those reviews (2014), additional systematic reviews of observational trials with meta-analysis have been published investigating the relationship between coffee consumption and CVD-related mortality, incidence/events and risk (Table 1). The findings from these trials largely agree with the umbrella reviews that coffee consumption is associated with cardioprotective traits. There have since been additional observational studies, notably data from the Brisihgella Heart study associating coffee consumption with reductions in peripheral and central BP [9] and results from the CLARIFY registry (coronary heart disease patients), showed no associations with key CVD outcomes [10].

Most studies have suggested there are potential benefits of coffee consumption on cardiovascular health, the associations commonly present in a non-linear relationship, with studies often reporting J-shaped or U-shaped curves in relation to dose and cardioprotection. This relationship suggests that low or no coffee consumption, as well as very high (typically over five cups), are not associated with beneficial vascular effects (and even detrimental effects); studies quote moderate coffee consumption for optimum benefits, approximately three–five cups per day [15,16].

It should be noted that observational trials do not prove causality nor can the direction of the relationship be determined. Robustly designed, randomized controlled trials (RCTs) are necessary to demonstrate this. Observational trials also come with other limitations associated with self-reporting dietary intake, such as reporting bias as well as recording limitations. For coffee, the latter is particularly important as there is no set definition as to what a cup of coffee is, e.g., the volume, how it is prepared and how it is consumed, which could prove important in understanding the relationships.

### 2.2. Evidence from Randomized Controlled Trials

Although there are a number of RCTs which use coffee as an intervention, very few focus on coffee as the whole beverage and are rather often used in modified, rather than commercially available coffee and focus on specific components within coffee such as caffeine or other bioactive compounds called polyphenols. These RCTs do not prove causality as they do not focus on the coffee beverage in its entirety.

On searching the literature, six RCTs were retrieved comparing coffee to a non-coffee control (Table 2). The trials all used markedly different interventions and had differing trial designs (different durations, populations, intervention amount) and hence it is difficult to draw sound comparisons. Only half of the trials retrieved were of a chronic nature, and in all, the sample sizes were small. Nonetheless, overall, three trials observed no impact of coffee on cardiovascular risk factors; in two, increases in blood pressure were observed and one noted a reduction in blood lipids. However, there is a significant need for well-designed RCTs focusing on key markers of CVD risk and/or CVD incidence to thoroughly test the observations in the prospective cohort trials and prove causality.

### 2.3. Interpretation of the Evidence for Coffee and Cardiovascular Health

There is strong evidence from observational trials that supports the notion that moderate coffee consumption is beneficial for cardiovascular health. However, this is not currently supported by evidence from RCTs, probably due to the limited number of well-designed trials to adequately answer that question, along with the heterogeneity in a cup of coffee. Therefore, the idea that coffee is beneficial to vascular health should not be rejected and warrants further, considered investigation. Below, we discuss the potential effects of the different bioactives in coffee and how they might impact the results observed.

## 3. Potential Mechanisms Driving Effects

**Figure 1:**
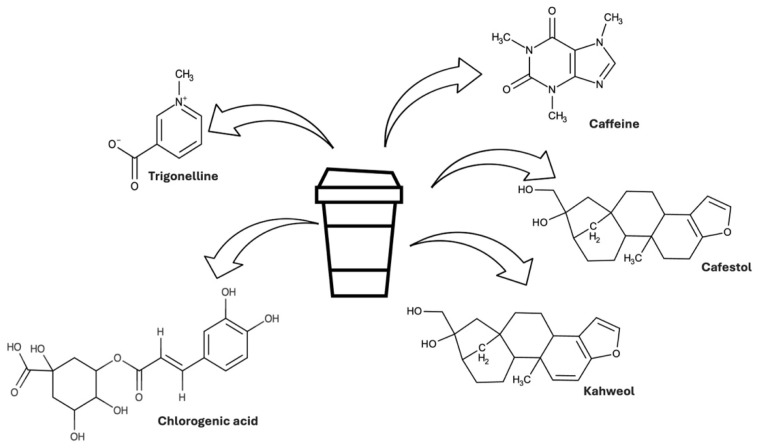
Examples of bioactive components in coffee.

Historically, interest in the impact of caffeine on cardiovascular health has dominated the research field. However, coffee is a chemically complex beverage containing thousands of chemicals, many of which have biological activity, including a range of phytochemicals with potential health effects. It is not clear which of these, or which combination of these, are driving the observed effects (Figure 1).

### 3.1. The Varying Levels of Bioactives in Coffee

The specific concentration of bioactive compounds in a coffee brew can vary dramatically depending on a number of factors. Due to the complexity of coffee, these experiments are difficult to control and hence published data are often inconsistent. Below, some of the elements that impact coffee bioactive content are summarized.

#### 3.1.1. Agricultural Factors

Pre-harvest considerations can play an important role in the content of coffee bioactives. The variety *Coffea arabica* (arabica) and *Coffea canephora* (robusta) are the varieties that currently dominate the commercial coffee market. Robusta has ~1.5 times more caffeine than arabica (as reviewed by Olechno et al. [23]); similarly, robusta also contains more CGA [24], while the variety contains lower levels of trigonelline [25]. The impact of variety on diterpene levels has been previously reviewed [26], and arabica beans seem to have higher diterpene levels, with robusta having much lower levels of kahweol compared to arabica.

Coffee beans grown at higher altitudes tend to have higher concentrations of CGA [27], caffeine and trigonellines [28,29]. Data on diterpenes are limited but suggest that coffee grown at higher altitudes has a lower diterpene content [29]. Shade-grown coffee tends to have higher levels of caffeine [30]. Lower CGA content seems to be seen in coffee grown in full sun versus that grown in shade [31]. The impact of shade on trigonelline levels is not clear [31].

#### 3.1.2. Commercial Processing Techniques

There are a number of commercial processing steps that can be employed in coffee production and the impact will depend hugely on the method employed. Of course, decaffeination removes caffeine but does not seem to have a profound impact on the levels of CGA, trigonelline or diterpenes [32,33,34]. The impact of roasting has been extensively researched, probably due to the importance of roasting on coffee flavor. It is well established that CGAs decrease on roasting [35,36,37,38], additionally trigonelline levels also deplete on roasting [39,40]. As it is thermally stable, caffeine levels are minimally impacted by roasting [39,41] and diterpenes are also not impacted by roasting [42].

Although coffee is commonly consumed in the UK, there are no available data on the impact of freeze- or spray-drying on the levels of bioactive content.

#### 3.1.3. At-Home Conditions

The way coffee is made at home can have a sizable impact on the levels of bioactive compounds. For example, the higher the water to coffee ratio, water temperature or time of brewing, the higher the extraction rate of water-soluble components, e.g., CGA and caffeine [43]. There is also evidence that high pressure and high temperature methods such as espresso extract more water-soluble components [44]. Boiling coffee not only produces brews with higher water-soluble components, but also results in higher diterpene levels [45]. When such coffee is not filtered, it results in much higher levels of diterpenes than the filtered counterpart [45]. Other commonly used preparation methods (e.g., filter coffee, espresso, moka pot, etc.) also impact the content of bioactives [46,47]. Notably, there is a lack of any comprehensive assessment of many of these factors and existing published data are heterogenous and hence it is very difficult to compare data between publications (e.g., when assessing the impact of coffee roasting, different bean varieties, preparation methods, bean origins, etc., may be used). This significant potential for variation in bioactive content means it is difficult to provide a definitive content of these compounds in coffee. Table 3 shows the range of content of bioactives analyzed in coffee brews, demonstrating the complexity of coffee research. This is particularly pertinent in relation to understanding the health impact of coffee as it could result in varied health effects; these variables are seldom considered in the context of observational trials. The impact of these individual components on cardiovascular health is discussed below.

### 3.2. Potential Drivers of Cardiovascular Health Effects in Coffee

**Figure 2:**
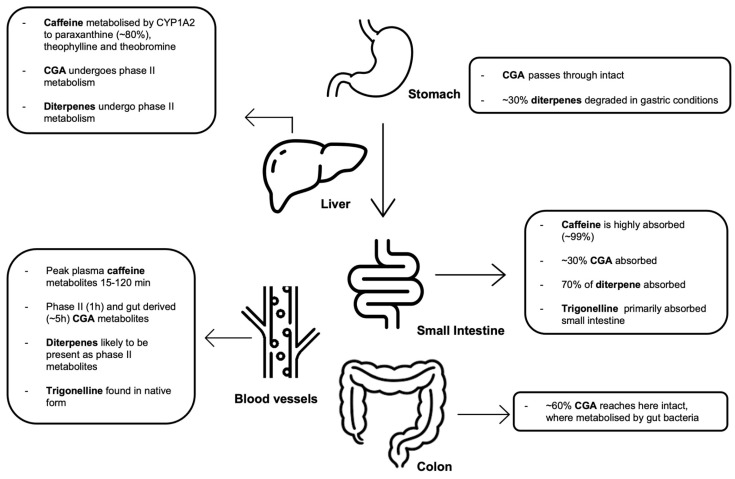
Metabolic pathways of caffeine, chlorogenic acid, diterpenes and trigonelline [40,58,59,81,82,83,94,111,112,117]. CGA is chlorogenic acid; min is minutes; h is hour.

The absorption, metabolism, vascular health effects and speculation about mechanisms of actions for the key coffee bioactives are discussed below. Figure 2 and Table 4 summarize these aspects.

#### 3.2.1. Caffeine

Caffeine is a methylxanthine and is the most recognized bioactive in coffee. It is the most widely consumed psychoactive substance internationally; an average cup of coffee contains about 75 mg of caffeine depending on the type and preparation method of the coffee; a moderate caffeine intake of up to 400 mg per day is considered safe for most adults [56]. Of the bioactives in coffee, caffeine is the most extensively researched.

##### Caffeine Absorption and Metabolism

Caffeine undergoes extensive metabolism in the liver cells, resulting in the formation of various compounds such as dimethylxanthines, monomethylxanthines, dimethyluric acids, monomethyluric acids and uracil derivatives. Phase I cytochrome (CYP) enzymes, particularly CYP1A2, are the primary enzymes involved in caffeine metabolism, accounting for approximately 13% of the total enzyme content in the human liver [57]; the CYP1A2 isoform is responsible for almost 90% of caffeine metabolism. Other enzymatic pathways involved in caffeine metabolism include CYP1A1, CYP2E1, CYP2A6, monooxygenase and N-acetyltransferase. Paraxanthine is the primary caffeine metabolite found in the plasma, while methylated xanthines and methyluric acids are the primary metabolites excreted in the urine [58]. Upon ingestion, caffeine is rapidly or almost completely absorbed into the bloodstream in the small intestine [59]. Caffeine can also be absorbed quickly through the oral mucosa, independently of the digestive system pathways. Studies have demonstrated that the time to reach peak plasma concentration after oral doses of 72–375 mg of caffeine varies between 15 and 60 min, with some cases of oral administration taking up to 120 min [57,60]. The speed of metabolism could, in part, be related to a polymorphism in the CYP1A2 gene [61].

##### Caffeine and Cardiovascular Health

The vascular impacts of caffeine have long been debated, a debate that has been exacerbated by the increase in consumption of high-caffeine energy drinks, especially amongst the young, which have been related to high profile cardiac-related deaths. It is well documented that caffeine impacts the vascular system, but the extent and nature of this impact is dependent on a number of variables. It should be noted that it is generally considered that consuming 400 mg per day is safe [56], although this level is different for certain populations (e.g., pregnant women).

It is likely that caffeine acts as an adenosine receptor antagonist. Caffeine and paraxanthine (a metabolite of caffeine) competitively bind to A1, A2 and other adenosine receptors [62], resulting in the blocking of adenosine inhibitory effects, and hence indirectly affecting the release of norepinephrine, dopamine, acetylcholine, serotonin, glutamate and gamma-aminobutyric acid (GABA) [48,63,64]. The result is mild central nervous system (CNS) stimulation, which is associated with the characteristic ‘wakening’ effects of caffeine and reported increased blood pressure. However, the impact on blood pressure is not consistently observed in trials, possibly due to other mechanisms impacting the vascular system, for example, increases in intracellular calcium in vascular smooth muscle cells and endothelial cells which can lead to increases in vasodilator nitric oxide (NO). However, there is evidence for reduced exhaled NO with caffeine, likely due to the aforementioned mechanisms [65].

The impact of caffeine on cardiovascular health continues to be debated, with varying outcomes observed, possibly due to the differing vehicles of delivery of caffeine (e.g., different types of coffee, caffeine tablets, energy drinks, etc.), food matrix, plus other components of the intervention could impact the outcomes.

A thorough review of 300 observational and experimental trials investigating the impact of caffeine intake on the body found moderate caffeine consumption (up to 600 mg/d) does not increase the risk of CVD, arrhythmia and heart failure in those who regularly consume caffeine. The acute increases in blood pressure are not considered to be sustained with regular consumption. Instead, it was suggested that moderate caffeine intake may reduce the risk of CVD in this population. Pre-hypertensive and hypertensive populations may experience acute blood pressure increases with caffeine intake at lower doses (up to 400 mg) and have a small increased risk of sustained hypertension [47].

A recent systematic review with meta-analysis demonstrated a non-linear relationship between caffeine and blood pressure (a key risk factor for CVD); however, though all doses demonstrated an increase in blood pressure, the confounding factors around the baseline blood pressure and level of habitual intake of caffeine should be considered [66]. This increase in blood pressure was also observed in a systematic review with energy drinks, along with reduced cardiac output. However, no other impacts on the vascular system were observed [67]. Interest in the impact of caffeine on endothelial function has recently emerged and is discussed in this recent review [68]; the authors concluded that caffeine can increase endothelial function but acknowledged the complexity of research in this field and highlight the need for further research.

#### 3.2.2. Chlorogenic Acids

Coffee is naturally high in polyphenols. In addition to minor contributors to the polyphenol content of coffee beans such as tannins, lignans and anthocyanins [69], the most abundant class in roasted coffee are hydroxycinnamic acid derivatives, usually termed chlorogenic acids (CGAs). They are esters of one or more hydroxycinamic acid with quinic acid. There have been at least 45 identified in coffee [70,71,72], with the most abundant being caffeoylquinic acids (CQAs), in which the hydroxycinnamic acid present is caffeic acid, dicaffeoylquinic acids (diCQAs), containing two caffeic acid moieties and feruyolquinic acids (FQAs), which contain ferulic acid. The most plentiful of these is isomer 5-caffeoylquinic acid, which is commonly simply termed CGA. This compound alone makes up approximately 35% of the total CGA in coffee [36]. A regular coffee consumer may consume in total 0.5–1 g of CGA per day [73].

##### Chlorogenic Acid Absorption and Metabolism

It is considered that CGA is not very bioavailable, with some studies unable to detect any in plasma [74] and others detecting low amounts of these native compounds [75,76,77,78]. The quinic acid moiety has been suggested as a contributing factor for the lack of intact absorption where seen [79]. It is likely that CGA avoids metabolism in the stomach due to its stability in acidic conditions and in digestive juice [74,80]. In fact, it is suggested that in the metabolism and absorption of CGA are minimal in the upper gastrointestinal tract, and in studies using effluent from ileostomy patients, it is believed that only approximately 30% of CGA absorption occurs before the colon [81,82]. Of the CGA not absorbed in the upper intestinal tract, it has been documented that approximately 78% has not yet undergone any metabolism at all [83]. The presence of enzymes such as cinnamoyl esterase in the small and large intestine could be responsible for the initial hydrolysis of the chlorogenic acid [74,84]. However, hydrolysis by enzymic means in the intestine is not always observed [85]. Further, bacteria in the colon may produce such enzymes and also contribute to the hydrolysis and subsequent metabolism of CGA, as has been demonstrated previously [84,85]. Hence, interindividual variation in gut microbiota composition could lead to differences in CGA metabolism.

There is an increasing amount of data with regard to the detailed fate of CGA in the body; however, due to the lack of commercial authentic standards for the metabolites found in vivo, many investigations just estimate plasma and urinary metabolites after enzymic hydrolysis quantifies only the free phenolic acids and CGA [86]. However, all of the work seems to agree that CGA metabolites are present in the plasma and urine predominantly as glucuronidated or sulfated phenolic acids. The work exploiting the use of authentic standards has identified 32 metabolites (including native CGA and free phenolic acids) in ileal effluent [83], and 22 metabolites from plasma and 10 from urine, which gives a much broader picture than could be obtained from a hydrolysis method alone as it allows for conjugates and isomers to be distinguished [77]. It is suggested that native CGA compounds and phenolic sulfates tend to reach their maximum plasma concentration at around 1 h, having been absorbed in the small intestine, and further metabolites which are produced in the colon peak in the plasma later at approximately 5 h post consumption, these are namely dihydrocaffeic and dihydroferulic acid and their sulfated counterparts [77]. On assessment of the CGA ADME (absorption, distribution, metabolism and excretion) profile post coffee consumption, 24 h urine collection indicated that CGA is more bioavailable than many of the other polyphenols, such as flavonoids and other phenolic acids [77].

##### Chlorogenic Acids and Cardiovascular Health

Compared to other polyphenol sub-classes, such as flavanols and anthocyanins, the evidence for CGA is limited. However, there is a growing body of evidence that CGA, like other polyphenols, plays a role in CVD prevention. Controlled human intervention studies involving coffee tend to focus on caffeine rather than on bioactive components such as CGA. Nonetheless, recent studies using flow mediated dilatation (FMD) to assess vascular function following coffee consumption have suggested that other compounds in coffee, such as CGA are capable of counteracting potential negative effects that caffeine has on the vascular system [78,87,88]. Additionally, there is evidence from randomized controlled trials supporting blood pressure lowering effects [89] as well as reduced arterial stiffness, as measured by cardio-ankle vascular index [87].

It is not known by which mechanism polyphenols, like CGA exert the observed beneficial effects. It is unlikely that they exert antioxidant capacity in circulation due to their extensive metabolism and minimal bioavailability [81]. It is more likely that the effects are nitric oxide mediated [90] and/or via modulation of the gut microbiota [91]. Work focusing on the mechanism is greatly needed in this field.

#### 3.2.3. Diterpenes: Cafestol and Kahweol

Cafestol and kahweol are natural diterpenes contained within coffee beans and present as fatty esters in unfiltered coffee [34,92]. They are released from the bean by the action of hot water and form a lipid layer in coffee. This lipid layer can be removed by paper filtration and is a common preparation step in most Western and European populations [93].

##### Diterpene Absorption and Metabolism

Research in ileostomy patients showed that the majority of ingested cafestol and kahweol (70%) enters the small intestine, where a small amount is excreted as conjugates of glucuronic acid or sulfate in the urine [94]. In mice, it is indicated that the majority of absorbed cafestol and kahweol is subject to more extensive metabolism, with evidence suggesting most accumulates in the liver and gastrointestinal tract via the enterohepatic cycle [95]. The rest is degraded in the gastric fluid before reaching the duodenum, and therefore, is not bioavailable [49]. In other human trials, differences in response to cafestol due to genetic polymorphisms of the apoE gene in normolipidemic subjects were observed, suggesting polymorphisms could influence metabolism [96].

##### Diterpenes and Cardiovascular Health

In 1983, findings from the Tromso heart study demonstrated a positive association between coffee consumption and blood cholesterol [97]; this was the seminal paper in this field. Since then, this relationship has been attributed to diterpenes present in unfiltered coffee and has been demonstrated numerous times [98,99,100,101,102,103]. Interestingly, an RCT that followed the Tromso observational data showed that filter coffee consumption did not significantly increase total cholesterol concentrations among consumers [104].

Although the exact mechanisms are yet to be determined, it is widely considered that cafestol is the compound that plays a key role in modulating cholesterol [98]. There is speculation from in vitro work that cafestol influences the gene expression of LDL receptors. In Caco 2 cells incubated with cafestol, an increase in uptake and degradation of LDL, potentially due to an increase in LDL receptor mRNA transcription and reduced secretion of cholesteryl ester and triacylglycerol [105]. Cafestol has been shown to reduce the binding, uptake and degradation of radiolabeled LDL, as well as a reduction in LDL receptor protein in human cell fibroblasts and HEPG2 cells [106].

Other proposed mechanisms include those involving lipid transfer proteins, cholesteryl ester transfer protein (CETP) and phospholipid transfer protein (PLTP). In RCTs, cafestol has been shown to increase the activity of these enzymes [99], and similar findings were observed in unfiltered French-press coffee versus filtered coffee [107].

#### 3.2.4. Trigonelline

Like caffeine, trigonelline is an alkaloid; it is a product of niacin metabolism. It is almost unique to coffee and therefore lends itself as a biomarker of coffee intake [108]. Unlike caffeine, trigonelline is not considered heat stable and is therefore partially decomposed to volatile pyridines and non-volatile N-methylpyridinium and nicotinic acid [109]. It is one of the lesser researched bioactive compounds in coffee, perhaps due to the low levels remaining in roasted coffee.

##### Trigonelline Absorption and Metabolism

Pharmacokinetics of trigonelline have been profiled in human participants after one and three cups of coffee [110]. The C_max_ and T_max_ were observed to be 2.33 and 6.13 µmol/L and 3.00 and 8.48 h, respectively, with observed sex-differences in absorption [110]. Details on the ADME for trigonelline in humans are lacking; most work has been conducted on animal models. It is considered that trigonelline is stable in the environment of the small intestine, the site of absorption [111], but some trigonelline reaches the large intestine intact. Data suggest trigonelline appears in circulation in its native form; to date, metabolites have not yet been reported [40,112].

##### Trigonelline and Cardiometabolic Health

There is limited evidence on the impact of trigonelline on health. In vitro assessment has demonstrated the antioxidant activity of trigonelline [113]. Necrosis and apoptosis were reduced up until a certain concentration, and other markers of oxidative stress were significantly affected [114]. This has been further demonstrated in diabetic rat models, in which an up-regulation of antioxidant activity and decreased lipid peroxidation were seen [115]. It has also been demonstrated in an animal model that trigonelline could modulate choline-metabolizing gut microbes, resulting in reduced choline and, hence, improvements in cardiovascular parameters [116].

**Table 4:** Overview of the vascular effects and mechanisms for caffeine, chlorogenic acid, diterpenes and trigonelline.

Bioactive	Potential Mechanism	Details	Reference
Caffeine	Adenosine receptor antagonist (blocks A1 and A2A receptors)	Increases blood pressure	[62]
	Increase intracellular calcium	Increases NO → vasodilation	[118]
Chlorogenic acid	Mechanisms largely unknown, antioxidant hypothesis dismissed		[81]
	Nitric oxide modulation	Inhibits NADPH oxidase → increases NO → vasodilation	[90]
	Gut microbiota modulation	Some evidence for beneficial modulation (e.g., increases in bifidobacteria) → increased SCFA → potential vascular benefits	[91,119]
	Reduce platelet aggregation	↓ Tromboxine A2 and ↑ cAMP and cGMP	[120]
Diterpenes (cafestol and kahweol)	Cholesterol regulation	↑ Serum lipid transfer protein → increased cholesterol	[99]
		↓ Bile acid synthesis → increase cholesterol	[121]
Trigonelline	Limited evidence for vascular effects		
	Gut microbiota modulation	↓ Choline metabolites → potential vascular benefits	[116]
	Antioxidant	↑ Antioxidant enzyme activity	[115]

NO is nitric oxide; SCFA is short chain fatty acid; cAMP is cyclic adenosine monophosphate; cGMP is cyclic guanosine monophosphate; ↑ is increase; ↓ is decrease.

## 4. Conclusions

Herein, we have discussed the current evidence from observational studies and RCTs on the impact of coffee consumption and cardiovascular health, avoiding conflation of causality with mechanism. We have coupled this with a detailed overview of the potential contribution to vascular effects by the key coffee bioactives.

Overall, the evidence suggests that coffee consumption is not detrimental to cardiovascular health, and indeed may play a role in CVD risk reduction and prevention. The association between moderate coffee consumption (three–five cups/day) and a reduced risk in CVD has been well documented in observational trials. However, a ‘cup of coffee’ is not defined and could vary dramatically in the level of bioactives as well as the nutritional composition (e.g., addition of cream and sugar) and hence pharmacological action. A comprehensive analysis of the impact of preparation method on bioactives is lacking and could contribute a more in depth understanding of the observational trials.

The evidence from observational trials does not translate to RCTs, likely due to the lack of RCTs testing the impact of coffee on CVD and disease risk and the heterogeneity of a cup of coffee. There is a need for some robust, long term RCTs to prove causality. The field would also benefit from trials to determine mechanisms, especially relating to the impact of bioactives, to support a full understanding of the role of coffee in CVD. There is some promising evidence that the observed beneficial effects may be due to the CGA content.

## Figures and Tables

**Table 1:** A summary of systematic reviews published since 2014 investigating associations between coffee intake and cardiovascular mortality, disease incidence or risk.

Reference	Year	N°^.^ Trials	Population	CVD-Related Findings
Ribeiro et al. [11]	2020	6	Post MI	Association with reduced CV mortality, no relationship with recurrent MI, stroke or MACE
Shahinfar et al. [12]	2021	10	T2D	4 cups/day associated with reduced CV mortality, CHD and CV events
Ding et al. [13]	2014	5	No exclusion	Association with reduced CV mortality in men (not women)
Di Maso et al. [14]	2021	26	No exclusion	Association with reduced CVD incidence and mortality

N°^.^ is ‘number of’; MI is myocardial infarction; CVD is cardiovascular disease; MACE is major adverse cardiac event; CHD is coronary heart disease.

**Table 2:** A summary of randomized controlled trials investigating the impact of whole coffee consumption on cardiovascular risk markers.

Reference	Year	Design	Duration	Population	Intervention	Control	Key Results
Rosmarin, et al. [17]	1990	Crossover	2 m	n = 24 healthy	Filter (mean 3.6 cup/d), caf	No coffee	No effect
Dusseldorp, et al. [18]	1991	Parallel	14 w	n = 64 healthy	Boiled +/− filter (6 cups/day), caf	No coffee	Boiled alone moderate increase in BP
Zhang, et al. [19]	2014	Crossover	3 h	n = 18 healthy	Freeze or spray dried instant (2 g), caf	Water	No effect
Ioakeimidis, et al. [20]	2017	Crossover	2.5 h	n = 24 healthy	Espresso (×3), caf or decaf	Water	Caf and decaf increased BP, caf increased AIx, PWV, AP and decaf only in non-habitual drinkers
Martínez-López, et al. [21]	2019	Crossover	8 w	n = 25/27 healthy/hypercholesterolemic	Instant, green blend, (6 g/day), caf	Water	Improvements in lipid profile in hypercholesterolemic
Lima de Castro, et al. [22]	2024	Crossover	1.5 h	n = 16 treated hypertensives	Pods (one), caf or decaf	Water	No effect

h is hour; d is day; w is week; m is month; caf is caffeinated; decaf is decaffeinated; BP is blood pressure; AIx is augmentation index; PWV is pulse wave velocity; AP is augmented pressure.

**Table 3:** Summary of major bioactive content of coffee brews.

Bioactive Compound	Dose Range Per Serving (mg) ^a^	References
Caffeine	45–310 mg	[48,49,50]
Total chlorogenic acids ^b^	50–445 mg	[36,48,51]
Cafestol	0–7 mg	[45,52,53]
Kahweol	0–7 mg	[45,52,53]
Trigonelline	38–540 mg	[47,54,55]

^a^ Dose has been adjusted to mg per cup, assuming a 200 mL cup for long coffee or 30 mL for espresso; ^b^ content as presented, usually the sum of the isomers quantified.
